# Bovinine

**Published:** 1888-09

**Authors:** 


### EDITORIALS AND MISCELLANEOUS.

BOVININE.—This is a fluid food prepared by the J. P. Bush Manufacturing Co., of New York and Chicago. It is highly recommended for its food and tonic properties in low states of the system. See the advertisement of this excellent preparation as found in this journal.

